# Epithelial–Mesenchymal Transition (EMT) 2021

**DOI:** 10.3390/ijms23105848

**Published:** 2022-05-23

**Authors:** Guidalberto Manfioletti, Monica Fedele

**Affiliations:** 1 Department of Life Sciences, University of Trieste, 34127 Trieste, Italy; manfiole@units.it; 2 National Research Council (CNR), Institute of Experimental Endocrinology and Oncology (IEOS), 80145 Naples, Italy

Epithelial–mesenchymal transition (EMT) is a transdifferentiation process wherein epithelial cells acquire characteristics typical of mesenchymal cells. In this dynamic and reversible transition, epithelial cells lose typical features such as junctions and baso-apical polarity while acquiring back-to-front polarity along with the ability to migrate and invade surrounding tissues. The high plasticity of the process is governed by epigenetics changes and mainly involves the modification of histones that regulate the expression of crucial EMT-transcription factors, a small cohort of master-regulators such as SNAIL, SLUG, TWIST, and ZEB that controls target key genes for EMT. These epigenetic and cellular dynamics occur during embryogenesis (Type 1 EMT), wound healing, tissue regeneration, and fibrosis (Type 2 EMT) and in cancer where they contribute to cell stemness, drug resistance, immune escape, and metastasis (Type 3 EMT); therefore, EMT is a field of active investigation that can have implications in both physiological and pathological processes.

The year 2021 has been a year of major breakthroughs in EMT covering different areas and human pathologies, including cancer and Coronavirus disease 2019. Among the most impactful contributions, we selected a few that are presented here.

The different stages of EMT, the so-called EMT continuum, and its reverse mesenchymal-epithelial transition (MET) are deeply involved in the phenotypic heterogeneity of cancer cells, which contributes to different hallmarks of cancer such as tumor invasion, metastasis, and chemoresistance. In this frame, during 2021, different studies demonstrated the relevance of the intermediate continuum of EMT states in metastatic progression. A genetic tracing system has been developed to monitor partial or full EMT of cells in the MMTV-PyMT mouse model of metastatic breast cancer [1]. Live imaging and 5-cell RNA-seq allowed the tracking of cells, showing that, in primary tumors, cancer cells that undergo partial EMT contribute to lung metastasis; instead, cells that have undergone a full EMT failed to colonize the lungs. To study cancer metastasis at high resolution, Simeonov et al. [2] developed a genetic system based on an inducible lineage tracer coupled with single-cell RNA-seq and applied it to an in vivo mouse model of pancreatic metastasis. Lineage reconstruction revealed that cells occupy a continuum of EMT states and that metastatic potential reaches the maximum in rare, late-hybrid EMT states. Moreover, the gene signature of these states are predictive of reduced survival in both human pancreatic and lung cancer patients. The induction of a hybrid EMT state is also the mechanism by which a loss of function of the *FAT1* gene promotes tumor initiation, progression, invasiveness, stemness, and metastasis [3].

The reciprocal influence between tumor and its microenvironment (TME) is critical in tumor progression and metastasis formation and can have implications in immunotherapy. To address this issue, the MMTV-PyMT breast carcinoma mouse model has been used to purify cancer cells with epithelial (E) and quasi-mesenchymal (qM) states. The analysis of these two cell populations showed that qM mammary carcinoma cells assemble an immunosuppressive TME and developed resistance to anti-CTLA4 immunocheckpoint blocking therapy, at variance from their more E counterparts, and that even a small number of qM cells can cross-protect E cells in the same tumor from immune attacks [4]. Authors identified cytokines secreted specifically by qM cells attracting protumor M2 tumor-associated macrophages (TAM) that play a pivotal role in influencing various immune cells in TME. Understanding the mechanisms by which qM cells resist immune therapy can help identify signalling pathways that can increase the efficacy of immunotherapies. On the same topic, another contribution supported the relevance of cancer cells in modifying TME, demonstrating that lung adenocarcinoma cells with high expression levels of the key EMT master-regulator ZEB1 activate a secretory program that reprograms cancer-associated fibroblasts (CAF) and drives them to the edge of the migratory front, thus governing CAF heterogeneity to promote metastasis [5]. Hence, cancer cells can not only influence TME but also the reverse is true. Combining single-cell RNA-seq from human glioblastomas and model systems with functional experiments, it has been demonstrated that macrophages induce a transition of glioblastoma cells into mesenchymal-like (MES-like) states [6]. Mechanistically, this effect is mediated by macrophage-derived oncostatin M that interacts with its receptor on glioblastoma cells and activates STAT3, thus inducing a MES-like state.

Using lung cancer models, Stewart et al. [7] revealed that infection with severe acute respiratory syndrome coronavirus 2 (SARS-CoV-2) induces EMT, which in turn causes the repression of the virus receptor ACE2 and contributes to the pathophysiology of Coronavirus disease 2019. The authors showed that once a cell is infected with SARS-CoV-2, there is a shift to a more mesenchymal phenotype characterized by high ZEB1 and AXL, putatively lower levels of miR-200, and a decreased dependence on glutamine synthesis. This opened an attractive therapeutic strategy based on the reversion of EMT with AXL inhibitors. Accordingly, AXL inhibitor gilteritinib was identified to have anti-viral activity against SARS-CoV-2 [8]

Epigenetic changes are crucial in governing EMT. Baggiolini et al. [9] found that the introduction of an oncogenic form of *BRAF* (*BRAF^V600E^*) in zebrafish during the neural crest and melanoblast stages efficiently developed tumors, whereas melanocytes were relatively resistant, and they could recapitulate these findings in a human pluripotent stem cells model. Gene expression analyses revealed the significant up-regulation of several chromatin-modifying enzymes in the more competent neural crest and melanoblast cells. One of these epigenetic modifiers, ATAD2, was shown to be specifically involved in controlling, together with SOX10, the expression of oncogenic and neural crest programs. On the same subject, Terranova et al. [10] found an association of *NRAS* mutants with specific histone modifications (H3K27me3) and the epigenetic repressor PRC2 in human melanomas. They demonstrate a reprogramming of these chromatin modifications on genes coding for EMT master transcription factors during melanoma metastasis and showed that a combination of PRC2 and MEK inhibitors markedly reduced tumor burden in a mouse xenograft model.

In this Special Issue, we have collected six articles (five original research studies and one review) showing how EMT is involved in different types of cancer, as well as in the metabolic dysfunction occurring in TGF-β2-induced retinal epithelial (RPE) cells and in metabolic reprogramming associated with tumor progression.

Riccioni and colleagues [11] show the role of hnRNP-Q (also known as SYNCRIP) as a positive modulator of EMT in hepatocytes, demonstrating that silencing of SYNCRIP induces MET, leading to inhibition of migration capacity of hepatocarcinoma cells. At the molecular level, they identify a panel of miRNAs, including miR-181-a1-3p, miR-181-b1-3p, miR-122-5p, miR-200a-5p, and miR-let7g-5p, which are regulated by SYNCRIP during EMT/MET dynamics. Armando et al. [12] characterized the EMT process of the equine penile cancer. The study is particularly interesting because human penile cancer is a neglected disease with no animal models for basic and pre-clinical research. Equine penile cancer may serve as a model for mimicking papillomavirus (HPV)-induced human lesions, so an in-depth characterization of this model is of potential interest not only to the veterinary community but also to the oncology/urology community. Related to HPV and EMT, low-grade ectocervical lesions have been investigated in the study by Ranieri and coworkers [13], who demonstrate that the E5 oncoprotein of HPV16 impacts the molecular profiles of EMT-related and differentiation genes. Colon carcinoma is instead the object of the study by Zhang et al. [14] reporting data on how BMAL1 knockdown influences the EMT/MET balance and what therapeutic consequences its downregulation confers. The latest research article by Shu et al. [15] highlights that TGFβ2 alters the structure and the respiratory capacity of mitochondria, thus reducing the production of ATP. Using ARPE-19 cells and primary RPE cells, they demonstrate that these alterations are linked to the reduced expression of PGC1α in cells treated with TGFβ2, supported, albeit indirectly, by the attenuation of the TGFβ2 action and, correspondingly, of its effects, upon the treatment of cells with ZLN005, a selective small molecule activator of PGC1α. The altered expression of metabolites and metabolic enzymes is central to our review article, which describes the complex interaction between EMT and metabolism during tumor progression. First, it outlines the main connections between the two processes, with particular emphasis on the role of cancer stem cells and LncRNAs. Hence, it focuses on specific cancers, such as breast, lung, and thyroid carcinoma [16].

We believe that this small collection of articles published in this Special Issue will be of interest for scientists working in the EMT field.

## Data Availability

Not applicable.
